# Gene expression profiling demonstrates a novel role for foetal fibrocytes and the umbilical vessels in human fetoplacental development

**DOI:** 10.1111/j.1582-4934.2008.00284.x

**Published:** 2008-02-25

**Authors:** Jung-Sun Kim, Roberto Romero, Adi Laurentiu Tarca, Christopher LaJeunesse, Yu Mi Han, Mi Jeong Kim, Yeon Lim Suh, Sorin Draghici, Pooja Mittal, Francesca Gotsch, Juan Pedro Kusanovic, Sonia Hassan, Chong Jai Kim

**Affiliations:** a Perinatology Research Branch, NICHD/NIH/DHHSBethesda, MD and Detroit, MI, USA; b Department of Pathology, Wayne State University School of MedicineDetroit, MI, USA; c Department of Obstetrics and Gynecology Wayne State University School of MedicineDetroit, MI, USA; d Center for Molecular Medicine and Genetics, Wayne State UniversityDetroit, MI, USA; e Department of Computer Science, Wayne State UniversityDetroit, MI, USA; f Department of Pathology, Sungkyunkwan University School of MedicineSeoul, Republic of Korea

**Keywords:** umbilical vein, umbilical artery, placenta, funisitis, chorioamnionitis, intrauterine growth restriction, transcriptome, microarray

## Abstract

There is a difference in the susceptibility to inflammation between the umbilical vein (UV) and the umbilical arteries (UAs). This led us to hypothesize that there is an intrinsic difference in the pro-inflammatory response between UA and UV. Real-time quantitative RT-PCR and microarray analysis revealed higher expression of interleukin (IL)-1β and IL-8 mRNA in the UV and differential expression of 567 genes between the UA and UV associated with distinct biological processes, including the immune response. Differential expression of human leukocyte antigen (HLA)-DRA mRNA between the UA and UV was due to unexpected HLA-DR^+^ cells migrating *via* the umbilical vessels into Wharton's jelly, more frequently in the UV. A significant proportion of these cells co-expressed CD45 and type I pro-collagen, and acquired CD163 or α-smooth muscle actin immunoreactivity in Wharton's jelly. Migrating cells were also found in the chorionic and stem villous vessels. Furthermore, the extent of migration increased with progression of gestation, but diminished in intrauterine growth restriction (IUGR). The observations herein strongly suggest that circulating foetal fibrocytes, routing *via* umbilical and placental vessels, are a reservoir for key cellular subsets in the placenta. This study reports fibrocytes in the human umbilical cord and placenta for the first time, and a novel role for both circulating foetal cells and the umbilical vessels in placental development, which is deranged in IUGR.

## Introduction

Establishment of an intra-amniotic infection induces a robust pro-inflammatory response in the mother, the foetus, and the placenta [1]. Acute funisitis, a component of acute chorioamnionitis, is the histologic hallmark of the foetal inflammatory response syndrome (FIRS) [2–4]. FIRS is a detrimental condition of the foetus characterized by elevated interleukin (IL)-6 in the foetal plasma and multi-organ involvement [5]. FIRS is associated with an increased risk of preterm delivery and both short- and long-term perinatal morbidities, such as chronic lung disease and cerebral palsy [6–9]. A key feature of funisitis is acute umbilical vasculitis. Interestingly, umbilical phlebitis almost invariably precedes umbilical arteritis [10, 11]. Although it has been shown that the endothelial cells of the umbilical vein (UV) may contribute to the differential inflammatory response by expressing more leukocyte adhesion molecules than those of the umbilical artery (UA) [12], the mechanisms responsible for the increased inflammatory response in the UV in comparison to the UAs remain to be elucidated.

Embryologically, two UAs arise from a distal branch of the dorsal aorta and two UVs develop as a pair of vessels, of which one disappears. As the liver develops, the right UV disappears and the persistent left UV becomes the single UV [13]. The UAs carry high-pressure but low-oxygenated blood from the foetus to the placenta, whereas the well-oxygenated placental blood travels through the UV to reach the foetal heart. After birth, the UAs constrict to prevent shunting of the neonate's blood to the placenta [14]. Smooth muscle and endothelial cells are known as the exclusive cellular components of both the UA and UV. The smooth muscle cells of the UV are found only in the media; the UA, on the other hand, has two layers of muscle coats in the intima and media [15]. Based on the structural differences between the UA and UV, and the progression patterns of inflammation, we hypothesized that there is an intrinsic difference in the biological response during the course of an inflammatory response between the UA and UV. A series of studies on gene expression and cellular immunopheno-types reported herein revealed that the umbilical vessels, especially the UV, are a novel migratory route for activated foetal fibrocytes. Recruitment of these multi-potential foetal cells into the Wharton's jelly and the placenta may be central to both fetoplacental development and response to insults.

## Material and methods

### Collection of umbilical vessels and explant culture

Segments of the UA and UV were collected from women in the following clinical groups: normal pregnancy at term not in labour (TNL; *n*= 15) and normal pregnancy at term in labour (TL; *n*= 15). Chorionic arteries and veins were also obtained from five normal pregnancies at term. All patients provided written informed consent, and the collection and use of the samples was approved by the Institutional Review Boards of the participating institutions.

Five pairs of the UA and UV from normal pregnancies at term were used for explant cultures. Segments of the UA and UV were placed in a 6-well plate and initially kept overnight in Dulbecco's Modified Eagle's Medium (DMEM) with Ham's F12 nutrient mixture (1:1) (Cellgro, Herndon, VA, USA) containing antibiotics and 10% foetal bovine serum, followed by further incubation in fresh media for an additional 24 hrs. The segments were then treated with lipopolysaccharide (LPS, *Escherichia coli* 0111:B4, L2630; Sigma, St Louis, MO, USA) at the concentration of 100 ng/ml for up to 6 hrs.

### Real-time quantitative reverse transcription-polymerase chain reaction (qRT-PCR)

Isolation of total RNA was performed using Trizol. DNase-treated total RNA was reverse transcribed using SuperScript III reverse transcriptase (Invitrogen, Carlsbad, CA, USA) and oligodT primers. qRT-PCR analyses were performed with TaqMan® Gene Expression Assays (IL-1β: Hs00174097_m1; IL-8: Hs00174103_m1; CXCL12: Hs00171022_m1; OR51E1: Hs00379183_m1; CREB5: Hs00191719_m1; NDUFA4L2: Hs00220041_m1; HLA DRA: Hs00219575_m1; NDUFA2: Hs00159575_m1; CCL13: Hs00234646_m1; Applied Biosystems, Foster City, CA, USA) using an ABI 7500 FAST Real Time PCR system. Human ribosomal protein, large P0 (RPLP0; Applied Biosystems) was used for normalization.

### Laser microdissection

Five-micrometre-thick frozen umbilical cord sections were obtained from five normal pregnancies at term and placed onto foil-covered glass slides (MicroDissect GmbH, Herborn, Germany). Using a Leica LMD6000 (Leica Microsystems, Wetzlar, Germany), endothelial layers and smooth muscle layers from the intima and media of the UA and UV were dissected. Total RNA in dissected samples was isolated using an RNeasy Mini kit (Qiagen, Valencia, CA, USA), followed by qRT-PCR analysis for IL-1β.

### Microarray analysis

Three segments (foetal, middle and placental) of the UA and UV were obtained from six normal pregnancies at term. Isolation of total RNA from umbilical vessels was performed using Trizol. An equal amount of the RNAs from the three segments was pooled for each UA and UV. After purification of RNA using an RNeasy Mini Kit, 500 ng of total RNA was amplified and biotin-labelled with the Illumina TotalPrep RNA Amplification kit (Ambion, Austin, TX, USA). Labelled cRNAs were hybridized to Illumina's Human-6 v2 expression BeadChips (Illumina, San Diego, CA, USA). BeadChips were imaged using a BeadArray Reader and raw data obtained with BeadStudio Software (Illumina). The complete data set is available in a MIAME (Minimal Information About a Microarray Experiment) complaint format in ArrayExperts (http://www.ebi.ac.uk/microarray-as/aer/) database (entry ID: E-TABM-368). Following quantile normalization of log-transformed intensity values, differential expression was inferred using a moderated paired two-sample t-test. The resulting *P*-values were adjusted using the false discovery rate method [16]. The differentially expressed genes underwent a functional analysis based on gene ontology (GO) terms using onto express [17, 18]. Canonical metabolic and signalling pathways from the Metacore® database (St. Joseph, MI, USA) were used to annotate the genes differentially expressed between the UA and UV. A pathway enrichment analysis was carried out using an over-representation approach available in the Metacore software. In addition, an impact analysis was performed on all signalling human pathways available in Kyoto Encyclopedia of Genes and Genomes (KEGG) with pathway express [19].

### Immunohistochemistry/Immunofluorescence staining

Umbilical cord specimens were obtained from women in the following clinical groups: (*i*) TNL (*n*= 15), (*ii*) TL (*n*= 15), *(iii)* intrauterine growth restriction at term (IUGR-T; *n*= 10), (*iv*) preterm labour/delivery (PTL; *n*= 10) and *(v)* intrauterine growth restriction at preterm (IUGR-P; *n*= 10). IUGR was defined as a birth weight below the fifth percentile for gestational age. Immunohistochemical staining was performed using a panel of antibodies to HLA-DRα (Dako, Carpinteria, CA, USA), CD14 (Vector Laboratories, Burlingame, CA, USA), CD68 (Dako), CD163 (Vector Laboratories), type I pro-collagen (Developmental Studies Hybridoma Bank at the University of Iowa, Iowa City, IA, USA), α-smooth muscle actin (α-SMA; AbCam, Cambridge, MA, USA), tryptase (AbCam), PU.1 (Santa Cruz Biotechnology Inc., Santa Cruz, CA, USA) and p65 NF-κB (RelA; Santa Cruz Biotechnology, Inc.). Automatic immunostainers (Ventana Discovery: Ventana Medical Systems, Inc., Tucson, AZ, USA) were used. DAB (3, 3′ -diaminobenzidine) and Vector Blue (Vector Laboratories) served as chromogens. The density of HLA-DR+ cells in the intramural and perivascular area of the UA and UV, respectively, was calculated as the cell number divided by the area measured using image analysis software (Image-ProPlus, Media Cybernetics, Silver Spring, MD, USA). The perivascular area was defined as that within one high-power field from the outer boundary of the vessel.

Immunofluorescence staining was performed using antibodies to CD34 (BD Biosciences Pharmingen, San Jose, CA, USA), HLA-DRα, CD45 (Dako), CD14, CD163, CXCR4 (AbCam), type I pro-collagen, and α-SMA. Secondary antibodies conjugated with Alexa fluor 488, Alexa fluor 594, Alexa fluor 555, FITC or Texas Red were used. The sections were examined using a Zeiss LSM510 confocal laser microscope (Zeiss, Jena, Germany) or a Leica TCS SP5 spectral confocal system (Leica Microsystems).

### Statistical analysis

The median mRNA expression for each cytokine and the median density of HLA-DR^+^ cells between the UA and UV were compared using a Wilcoxon signed-rank test. Kruskal–Wallis H and Mann-Whitney U tests were used to determine the differences in the median mRNA expression for each cytokine and the median density of HLA-DR^+^ cells among groups. SPSS version 12.0 (SPSS Inc., Chicago, IL, USA) was employed for statistical analysis. A *P*-value of <0.05 was considered statistically significant.

## Results

### Differential expression of IL-1β and IL-8 mRNA and anatomical constitution of the UA and UV

**Fig. 1:**
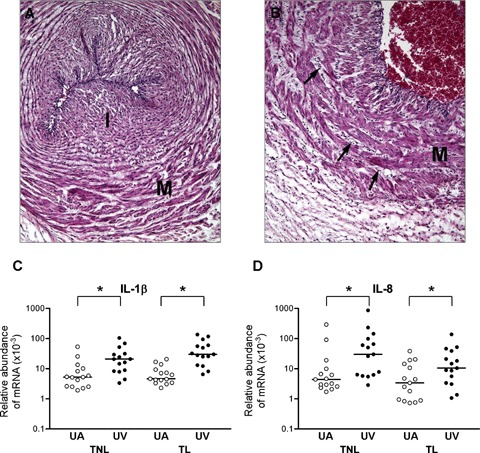
Differential interleukin (IL)-1β and IL-8 mRNA expression in umbilical artery (UA) and umbilical vein (UV). (**A, B**) UA (**A**) and UV (**B**) differ in the arrangements of muscle coats; UA has two smooth muscle layers in the intima (I) and media (M). Intimal smooth muscle cells are longitudinally oriented, while medial smooth muscle cells are circularly arranged. Neutrophilic infiltration is evident in the UV (arrows), but not in the UA in the same umbilical cord. Original magnification ×100. (**C**, **D**) mRNA expression of IL-1β (**C**) and IL-8 (**D**) was higher in the UV than in the UA in women at term not in labour (TNL) and term in labour (TL). *P < 0.05.

**Fig. 2:**
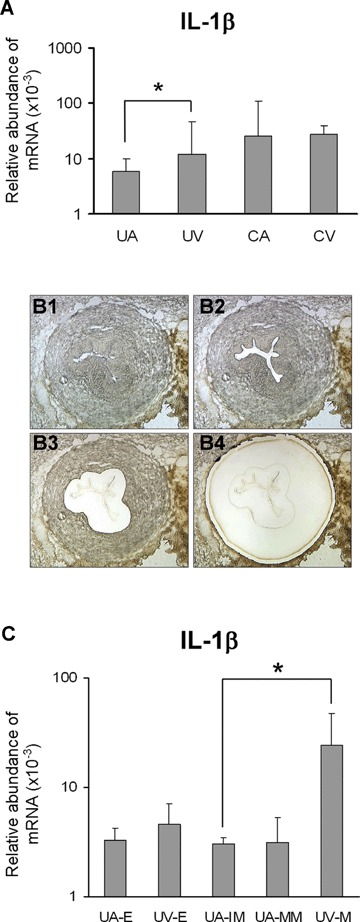
Evidence for an association of gene expression levels with intrinsic anatomical differences between the UA and UV. (**A**) Differential expression of IL-1β between umbilical and chorionic vessels. In contrast to a higher expression of IL-1β mRNA in the UV than that in the UA of the same cases from normal deliveries at term, there was no significant difference between chorionic arteries (CA) and veins (CV). *P< 0.05. (**B**) B1-B4 shows sequential images obtained during laser microdissection of an umbilical artery. B1: before microdissection; B2: after microdissection of the endothelial cell layer; B3: after microdissection of the intimal smooth muscle layer; B4: after microdissection of the medial smooth muscle layer. Original magnification ×50. (**C**) The IL-1β mRNA was expressed higher in the smooth muscle layer of the UV (UV-M) than in the intimal smooth muscle layer of the UA (UA-IM), whereas there was no difference in the endothelial cell layer between the UA (UA-E) and UV (UV-E). There was no difference in the smooth muscle layer between the intima (UA-IM) and media of the UA (UA-MM). *P< 0.05.

For the initial screening of potential differences in the pro-inflammatory response, we compared the mRNA expression of cytokines IL-1β, a principal cytokine involved in pro-inflammatory response of leukocytes, and IL-8, a major chemokine for neu-trophils, in the UA and UV obtained from women at term by qRT-PCR. mRNA expression of both IL-1β and IL-8 was higher in the UV than in the UA, both in TNL and TL cases *(P<* 0.05, respectively) (Fig. 1). To further address whether the differential pro-inflammatory response of umbilical vessels results from differences in the constitution of umbilical arterial and venous blood, IL-1β mRNA expression was compared in both chorionic and umbilical vessels of term cases. Of interest, there was no difference between IL-1β mRNA expression in chorionic arteries and veins (P > 0.05), while it was higher in the UV than in the UA of the same placentas (P < 0.05) (Fig. 2A). These findings strongly suggest that intrinsic anatomical differences are a likely explanation for the differences in gene expression observed between these two vessels.

As the UA and UV are composed of an endothelial layer and smooth muscle layers, the differential gene expressions could be due to either a difference between the endothelial layers or between muscle layers. To determine the anatomical layer responsible for the differences in cytokine gene expression, IL-1β mRNA expression was evaluated in the endothelial layer and smooth muscle layers of the UA and UV using laser microdissected samples. The muscle layers of the arterial intima and media were analysed individually. qRT-PCR analysis revealed higher IL-1β mRNA expression in the UV muscle layer than in the intimal muscle layer of the UA (*P* < 0.05), whereas there was no difference in IL-1β mRNA expression between the endothelial cell layers of the UA and UV. There were no differences in expression for this cytokine between the intimal and medial muscle layers of the UA (Fig. 2B and C). These results also supported a role for anatomical differences between UA and UV in the differential expression of the cytokine genes tested.

**Fig. 3:**
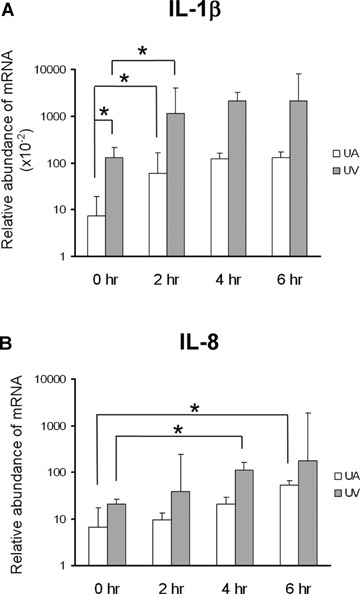
Induction of cytokines in umbilical vessel explants by lipopolysaccharide (LPS). The increase of IL-1β mRNA expression in the UA and UV following LPS treatment preceded that of IL-8. (**A**) IL-1β mRNA expression in both the UA and UV was increased significantly 2 hrs after LPS treatment. (**B**) IL-8 mRNA expressions in the UV and UA were increased 4 and 6 hrs after LPS treatment, respectively. *P < 0.05.

We also examined at the regulation patterns of both cytokines (IL-1β and IL-8) in the UA and UV explants following LPS treatment. The mRNA expression of IL-1β before LPS treatment was higher in the UV than in the UA (*P* < 0.05). LPS treatment increased the IL-1β and IL-8 mRNA expression in both the UA and UV (*P* < 0.05). Of note, IL-1β response following LPS treatment, which was evident after 2 hrs, preceded that of IL-8 in both vessels (Fig. 3A). A significant increase in IL-8 mRNA expression in the UV and UA was observed 4 and 6 hrs after LPS treatment, respectively (Fig. 3B).

### Transcriptome profiling of the UA and UV: enrichment of immune response among differentially regulated genes

**Fig. 4:**
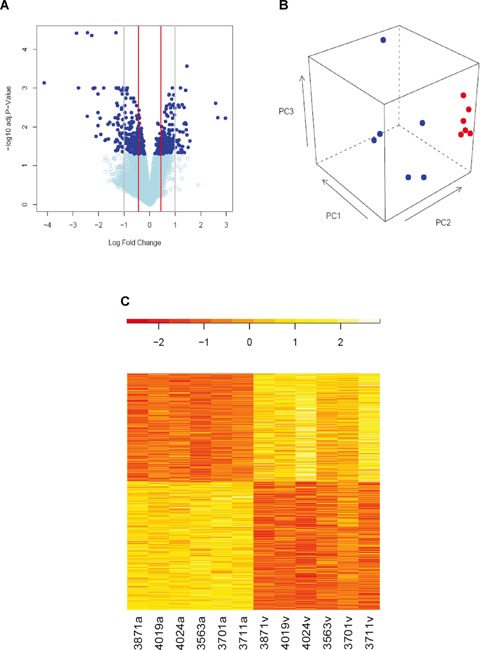
Gene expression profile of UA and UV by microarray analysis. (**A**) A volcano plot showing the relationship between the fold changes and false discovery rate (FDR) corrected *P*-values of the genes. Dots outside the two grey lines have a fold change greater than 2, while dots outside the two red lines have a fold change greater than 1.35, which is the detection limit claimed by the manufacturer. Positive log fold change values indicate that the expression in UV samples is higher, while negative values show the opposite. Filled blue circles designate genes with FDR < 0.05. (**B**) A principal component analysis plot generated with data from six pairs of samples (six UA and six UV), showing a clear segregation of the UA (red dots) and UV (blue dots). (**C**) The expression patterns of the 567 genes in the UA and UV are shown in a heat map. Rows correspond to genes while columns correspond to samples. High expression levels are shown in yellow while low expression levels are in red.

With the solid evidence for the differential expression of IL-1β and IL-8 mRNAs between the UA and UV, we extended the study of gene expression using microarray to identify global differences in the transcriptomes of the UA and UV. A comparison of six pairs of UAs and UVs obtained from normal pregnancies at term revealed differential regulation of 567 genes out of 20,720 annotated genes on the array (*P* < 0.05). qRT-PCR confirmed the differential expression of five genes (CXCL12, OR51E1, CREB5, NDUFA4L2, HLA-DRA) among those identified by the microarray analysis (*P* < 0.05). qRT-PCR analysis of two non-differentially expressed genes (NDUFA2, CCL13) also yielded results consistent with microarray analysis (data not shown). The results of microarray profiling are shown in Fig. 4. The ‘volcano plot’ (Fig. 4A) shows the magnitude of differential gene expression and demonstrates the level of significance. A principal component analysis was performed (as described previously [20]) to reduce the dimensionality of the gene expression data from a large number of genes (dimensions) to only three dimensions (principal components), so that we could visualize the samples in a 3D plot. It showed a clear distinction between the UA and UV based on the expression values of all genes monitored (Fig. 4B). The expression levels of the 567 genes are shown in Fig. 4C.

**Fig. 5:**
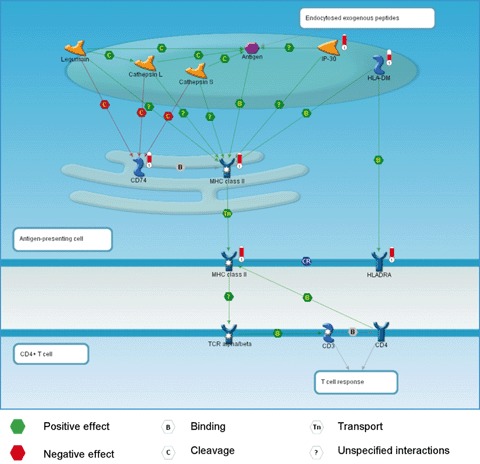
Differential gene expression involved in the pathway of antigen presentation by MHC class II. A diagram showing the pathway of antigen presentation by MHC class II, which was most significantly associated with the differential gene expression between the UA and UV. The genes expressed higher in UV than those in the UA are indicated by red bars.

GO analysis revealed over-representation of distinct biological processes between the UA and UV (Table 1). The following biological processes were enriched: N-acetylglucosamine metabolism, immune response, response to wounding, organ morphogenesis, apoptosis and smooth muscle contraction. The analysis of the metabolic and signalling pathways contained in the Metacore database yielded five pathways significantly associated with the condition under study (Table 2). These pathways are antigen presentation by major histocompatibility complex (MHC) class II (Fig. 5), reverse signalling by ephrin B, CXCR4 signalling pathway, immunological synapse formation and Notch signalling pathway. The impact analysis using pathway express identified two highly impacted pathways: *(i)* cell adhesion molecules and *(ii)* antigen processing and presentation.

**Table 1:** A list of differentially expressed genes associated with enriched biological processes in umbilical vessels *(P* < 0.05)

Genes overexpressed in UA			Genes overexpressed in UV		

N-acetylglucosamine metabolism					

CHST7			CHST6	LARGE	
**Immune response**					
CXCL12	NOX4	IFIH1	OSM	ZFP36	IFI30
NKX2–3	INHBB	TNFAIP6	BDKRB1	AQP9	LILRB3
GPR68	GEM	AGT	SEMA4D	HLA-DOA	KLF6
IFI6	UACA	GBX2	NDST1	C3	CD74
JAG2	IFIT2	CD55	WAS	IL1B	TLR5
MAPRE2 ULBP1	IL1RAP	CFH	HLA-DRA	CXCR4L	FPR1
			NFATC4	HLA-DMB	ALOX5
			OLR1	Y86	IGSF6
			CXCL16	HLA-DPA1	HLA-DMA
			ANXA11		

**Response to wounding**					

CXCL12	NOX4	F2R	ZFP36	BDKRB1	F5
TNFAIP6	GPR68	PDGFB	NDST1	C3	CD74
UACA	SOX15	MAPRE2	WAS	NINJ2	IL1B
PLSCR4 ADM	IL1RAP	F10	TLR5	CXCR4	FPR1
			ALOX5	NFATC4	OLR1
			LY86	CXCL16	HBEGF

**Organ morphogenesis**					

VANGL2	EFNB2	INHBB	FGF9	ALDH1A2	ADAMTS1
ODAM	AGT	ENPEP	SEMA6A	ANPEP	ITGA7
EDG1	JAG1	GBX2	BHLHB3	CXCR4	ABLIM1
JAG2	SOX15	ANGPTL4	DLL1	EDNRA	NFATC4
SHROOM2	ADM	VEGFC	ID1	DCN	
TBX1	NME2	COL18A1			
NRP1					

**Wnt receptor signalling pathway**					

FRZB	DKK2	WISP1	AXIN2		
FZD2	WNT2B	SLC9A3R1			

**Embryonic development (sensu Mammalia)**					

PTPRR			DLL1	EDNRA	

**Apoptosis**					

IFIH1	F2R	AGT	OSM	AXUD1	SEMA6A
IFI6	CTNNAL1	JAG2	SEMA4D	P2RX1	PSEN2
HSPE1	ANGPTL4	TOP2A	CARD9	CD74	IL1B
SULF1	CDC2	NUPR1	ACTN1	CXCR4	PIM1
EGLN3	MTP18		PTK2B	ADAMTSL4	KCNIP3
			IER3	LY86	

**Inactivation of MAPK activity**					

			DUSP8	DUSP2	

**Behaviour**					

CXCL12	EFNB3	KAL1	TRPV1	OSM	ITGA5
PTGDS	AGT	EPHA4	GNA01	FOSB	IL1B
CMTM8	CNR1	LEP	CXCR4	FPR1	CXCL16

**Smooth muscle contraction**					

NMU			CNN1	SMTN	EDNRA

**Table 2:** A list of pathways associated with differentially expressed genes between UA and UV by over-representation analysis using Metacore software

Pathway name	Corrected *P*-value	Number of differentially expressed genes/number of total genes
Antigen presentation by MHC class II	0.0006	5/12
Reverse signalling by ephrin B	0.0006	7/32
CXCR4 signalling pathway	0.0062	6/33
Immunological synapse formation	0.0199	7/61
Notch signalling pathway	0.0199	5/29

### Circulating foetal fibrocytes route into the Wharton's jelly through umbilical vessels: a physiological reservoir for macrophages and myofibroblasts

**Fig. 6:**
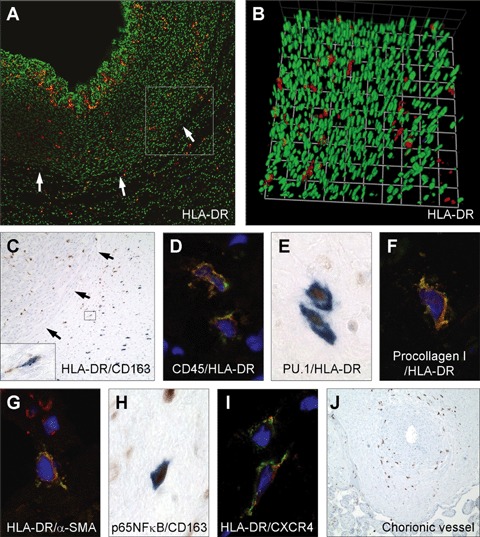
Immunophenotypes of migrating fibrocytes in the umbilical cord. (**A, B**) HLA-DR^+^ cells (red) migrate through the vascular wall into the Wharton's jelly of the umbilical cord. **B** is a z-stack image of a square shown in (**A**). All of the nuclei were stained with sytox green (green). Arrows indicate the boundary between the UV and Wharton's jelly. (**C**) HLA-DR (brown) and CD163 (blue) double immunoreactive cells are found in the outer layer of the vessel wall and in the adjacent Wharton's jelly. Most HLA-DR single positive cells are found in the vessel wall, while the majority of CD163 single positive cells are in the Wharton's jelly. The inset is a higher magnification of the rectangular area. Arrows indicate the boundary between the UV and Wharton's jelly. (**D**) CD45 expression in HLA-DR^+^ cells (CD45, green; HLA-DR, red; double positive, yellow). (**E**) Nuclear labelling of PU.1 (brown) in HLA-DR (blue) positive cells. (**F**) Co-expression of type I pro-collagen in HLA-DR^+^ cells (Type I pro-collagen, green; HLA-DR, red; double positive, yellow). (**G**) Co-expression of α-smooth muscle actin (α-SMA) in HLA-DR cells (HLA-DR, green; α-SMA, red; double positive, yellow) (**H**) Nuclear labelling of p65 NF-κβ (brown) in CD163^+^ cells (blue). (**I**) CXCR4 expression in HLA-DR^+^ cells (HLA-DR, green; CXCR4, red; double positive, yellow). (**J**) HLA-DR^+^ cells in and around the chorionic vessel of the chorionic plate. Original magnifications ×20 (**A, B**), ×100 (**C, J**), ×40 (**D, F, G**), ×1000 (**E, H**), ×63 (**I**).

As the genes related to antigen presentation by MHC class II were found to be the most differentially expressed in the UA and UV, HLA-DR immunostaining was performed. We found a novel population of HLA-DR^+^ cells routing through the umbilical vessels into the Wharton's jelly (Fig. 6A and B). Most cells were positive for CD68 and showed gradual increase in CD163 and CD14 immunoreactivity as they reached the outer layer of the vessel wall and the Wharton's jelly (Fig. 6C). HLA-DR immunoreactivity, on the other hand, gradually decreased at the peripheral region of the Wharton's jelly, showing an inverse relationship with that of CD163. The migrating cells were positive for CD45 (Fig. 6D), and showed a frequent nuclear labelling of PU.1 (Fig. 6E), indicating their haematopoietic origin [21]. These cells were negative for CD34 (data not shown). Of interest, type I pro-collagen and α- SMA expression were readily detected in a significant proportion of these cells (Fig. 6F and G), and the nuclei were positive for p65 NF-κB (RelA) (Fig. 6H). They were also strongly positive for CXCR4, another gene found to be differentially expressed between the UA and UV (Fig. 6I). Collectively, the findings indicated that these cells are haematopoietic in origin and that they have the potential for antigen presentation and fibroblast/myofibroblast differentiation, features consistent with those which have been described for fibrocytes [22]. An identical population of HLA-DR^+^ migrating cells was found in and around chorionic vessels and stem villous vessels (Fig. 6J).

**Fig. 7:**
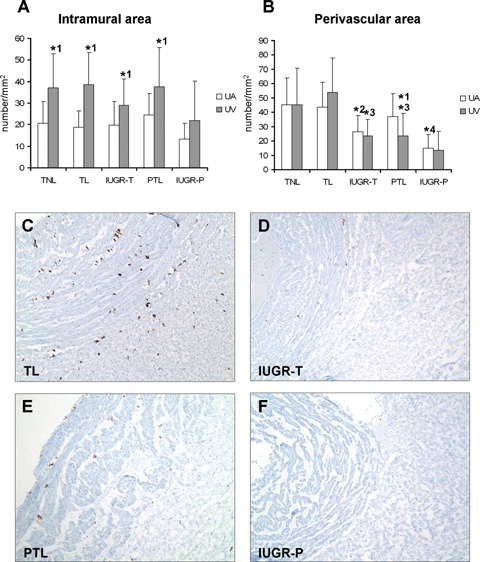
HLA-DR^+^ cell density in the umbilical cord among clinical groups. (**A**) The density of HLA-DR^+^ cells is higher in the UV than in the UA of term in labour (TL), term not in labour (TNL), intrauterine growth restriction at term (IUGR-T) and preterm labour (PTL) cases. (**B**) HLA-DR^+^ cells were found less frequently in the perivascular Wharton's jelly surrounding the UV of PTL cases than that of TL. IUGR cases at term (IUGR-T) and preterm (IUGR-P) showed a lower density of HLA-DR^+^ cells in the perivascular Wharton's jelly than the gestational age-matched non-IUGR cases (TL, TNL and PTL) (*1: *P* < 0.05 in comparison to the UA of the same group; *2: *P* < 0.05 in comparison to the same vessel of TNL; *3: *P* < 0.05 in comparison to the same vessel of TL; *4: *P* < 0.05 in comparison to the same vessel of PTL). (**C-F**) The panels show the representative migration patterns and extent of HLA-DR^+^ cells in the umbilical vein and perivascular Wharton's jelly of TL (**C**), IUGR-T (**D**), PTL (**E**) and IUGR-P (**F**). Original magnification ×100 (**C–F**).

Finally, the density of HLA-DR^+^ cells was compared according to gestational age and diagnostic group to determine the relationship between these factors/clinical diagnosis and the extent of HLA-DR^+^ cell migration. Consistent with the microarray results, HLA-DR^+^ cells were found more frequently in the UV than in the UA of the placentas irrespective of clinical conditions (Fig. 7A). A comparison of HLA-DR^+^ cell density in the perivascular Wharton's jelly revealed a higher density in the area surrounding the UV of cases in labour at term than in those with preterm labour/delivery *(P* < 0.05), suggesting a gestational age-dependent increase in the migration (Fig. 7B). Furthermore, perivascular HLA-DR^+^ cell density was significantly lower in IUGR cases compared to that of the gestational age-matched non-IUGR cases (Fig. 7B-F).

## Discussion

The principal findings of this study are: (1) The UV shows a greater inflammatory response than the UA, as a part of differences in global gene expression; (2) Genes involved in the immune response are among those enriched as determined by GO between the UA and UV (based upon microarray analysis); (3) HLA-DR^+^ foetal fibrocytes migrate *via* umbilical vessels into the Wharton's jelly and (4) The extent of fibrocyte migration increases as a function of gestational age while it is decreased in IUGR.

The increased pro-inflammatory response of the UV over that of the UA is in contrast to previous observations in other vessels, where arterial pro-inflammatory responses are dominant. Pro-inflammatory cytokine expression in the smooth muscle of adult coronary arteries is higher than that of the saphenous vein [23]. In addition, the expression of a subset of anti-inflammatory genes was also found to be higher in the saphenous vein endothelial cells than in those of the coronary artery [24]. Several comparisons of vascular transcriptomes – either endothelial cells or smooth muscle cells – in humans have been reported [23–26]. However, comparative gene expression profiling of the UA and the UV have not been reported thus far. We show that the UA and UV differ by the molecular signatures of more than 560 genes. Moreover, the biological processes revealed by GO and pathway analysis are functionally relevant. For example, in the case of smooth muscle contraction, the UA is rapidly closed due to muscular contraction initiated immediately following delivery [14]. The closure begins as a localized contraction, called ‘folds of Hoboken’[27, 28], which then spreads to the remainder of the vessel. The closure of the UA after delivery is crucial to prevent shunting of the oxygenated blood from the neonate to the placenta. Enrichment of genes involved in the immune response strongly suggests that the UA and UV are immunologically distinct and active. All of the other pathways associated with differentially expressed genes between the UA and UV, antigen presentation, CXCR4 signalling pathway and immunological synapse formation, are related to immune response, besides those related to arterio-venous differentiation, ephrinB and Notch signalling pathways. The most enriched biological process found in umbilical vessels, N-acetylglucosamine metabolism, is also relevant to immune response because N-acetylglucosamine is not only one of the key epitopes of microorganisms, but also a basic structural component of most molecules involved in the innate and adaptive immune response, such as immunoglobulins, T cell receptors and collectins [29]. The earlier involvement of the UV in acute funisitis and the extent of HLA-DR^+^ cell migration indicate that differential gene expression between the UA and UV is closely associated with both physiologic and pathologic migratory responses of circulating foetal leukocytes.

The phenotype of the HLA-DR^+^ cells reported here is unique. The cells express CD45, a haematopoietic cell-specific transcription factor PU.1, CXCR4, monocyte/macrophage antigens such as CD14, CD68 and CD163, and characteristically type I pro-collagen. Myofibroblast differentiation in the Wharton's jelly is also evident. Recent studies have reported primitive cell populations derived from circulating monocytes with the capacity to differentiate into non-phagocytic cells [30–32]. The fibrocytes share characteristics of haematopoietic stem cells and mesenchymal stem cells; they not only express several haematopoietic cell surface markers such as CD34, CD45 and HLA-DR, but also mesenchymal cell markers such as collagen and α-SMA [22]. Fibrocytes have been shown to play an important role in wound repair, granuloma formation, antigen presentation, vascular remodelling and various fibrosing disorders. They produce large quantities of extracellular matrix components, secrete growth factors and cytokines, and have antigen presenting properties [22]. Monocyte-derived multipotential cells (MOMCs) share an almost identical immunophenotype with fibrocytes but have the capacity to differentiate into a variety of mesenchymal cells [33]. Although the expression of CD34 was not readily detected, the HLA-DR^+^ migrating cells displayed an immunophenotype (CD45^+^/CD14^+^/CD163^+^/type I pro-collagen^+^) highly consistent with that of fibrocytes: the collagen-expressing monocytes. Cells in the Wharton's jelly have been reported as mesenchymal stem cells with a potential for differentiation into adipocytes, osteoblasts, chondrocytes, cardiomyocytes and neurons [34–36]. It is uncertain whether they represent a different group of cells, or the migrated fibrocytes or MOMCs that we found. Fibrocytes express several chemokine receptors, including CXCR4, whose ligand is CXCL12 [37]. Therefore, the higher expression of CXCR4 in the UV is relevant and we confirmed CXCR4 expression in HLA-DR^+^ cells in the umbilical vessel. Interestingly, CXCL12 expression was higher in the UA than in the UV. CXCL12 recruits fibrocytes in a murine model of pulmonary fibrosis [38], but not in a model of wound repair [37]. It is suggested that CXCL12/CXCR4 interaction is important, especially in the injury-related stem cell recruitment after myocardial infarction, whereas CXCL12 action alone seems to be insufficient for mobilization of CXCR4 expressing cells in physiological situations [39].

**Fig. 8:**
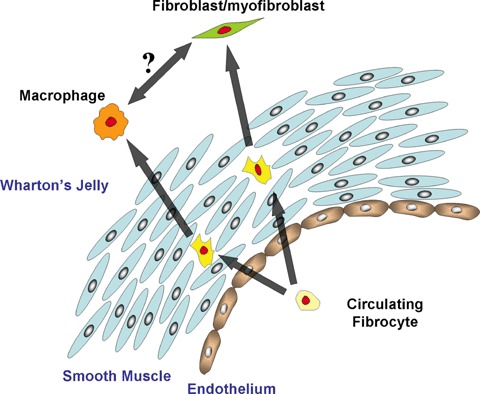
A proposed model of foetal fibrocyte migration and differentiation in the umbilical cord. The major changes in immunopheno-typic profiles of HLA-DR^+^/type I pro-collagen **+** fibrocytes migrating through the umbilical vessel are a decrease in HLA-DR and increased expression of CD163 or α-SMA. The fibrocytes differentiate into the macrophages or myofibroblasts, which are the main population of cells in the Wharton's jelly. It is uncertain whether there is a transdifferentiation sequence between the macrophages and myofibroblasts (?).

Fibroblasts/myofibroblasts form a major proportion of placental connective tissue and macrophages and mast cells are the only subsets of immune cells normally found in the placenta [40]. Migration of fibrocytes out of chorionic vessels and stem villous vessels strongly suggests that these cells are important for the development of the placental stromal mass. Gestational age-dependent changes in the extent of migration and the perturbation of migration with IUGR provide further evidence in support of this concept. IUGR is often associated with a macroscopically thin, fibrotic umbilical cord with a decreased amount of the Wharton's jelly matrix and a small placenta [41, 42]. Sequential changes in immunophenotype with migration into the Wharton's jelly and the differentiation of the cells are displayed in Fig. 8. It is of note that CD163^+^/CD14^+^ but HLA-DR^”^ macrophages are encountered in the Wharton's jelly, suggesting defective antigen presenting function. HLA-DR negative or dim monocytes/macrophages have been reported in sepsis or cancer patients [43, 44], and the biological relevance of decreased HLA-DR expression in some Wharton's jelly macrophages needs further study. In the context that many of these migrating cells are CD163^+^ with nuclear labelling for p65 NF-κB and that the extent of their migration increases with the progression of gestation, it is noteworthy that surfactant protein-A (SP-A)-induced NF-κB activation of the macrophages in the amniotic fluid at term is a key event initiating labour in mice. The activated macrophages migrate into the uterine wall where increased production of IL-1β leads to uterine contraction and parturition [45].

Differential gene expression profiles between the UA and UV are part of the mechanisms by which the progression pattern of acute funisitis can be accounted. In the present study, we report a yet unknown way of how the biological responses of the foetal umbilical/placental vessels and circulating foetal cells are integrated with placental development. This study also provides a list of potentially important target genes and biological processes for further investigation of human pregnancy and development.
